# CD8^+^CD161^+^ T-Cells: Cytotoxic Memory Cells With High Therapeutic Potential

**DOI:** 10.3389/fimmu.2020.613204

**Published:** 2021-02-01

**Authors:** Vanaja Konduri, Damilola Oyewole-Said, Jonathan Vazquez-Perez, Scott A. Weldon, Matthew M. Halpert, Jonathan M. Levitt, William K. Decker

**Affiliations:** ^1^ Department of Pathology & Immunology, Baylor College of Medicine, Houston, TX, United States; ^2^ Michael E. DeBakey Department of Surgery, Baylor College of Medicine, Houston, TX, United States; ^3^ Dan L. Duncan Cancer Center, Baylor College of Medicine, Houston, TX, United States; ^4^ Scott Department of Urology, Baylor College of Medicine, Houston, TX, United States; ^5^ Center for Cell and Gene Therapy, Baylor College of Medicine, Houston, TX, United States

**Keywords:** KLRB1, CD161, effector memory, T-cell, T_H_1 polarization

## Abstract

NK1.1 and its human homolog CD161 are expressed on NK cells, subsets of CD4^+^ and CD8^+^ T cells, and NKT cells. While the expression of NK1.1 is thought to be inhibitory to NK cell function, it is reported to play both costimulatory and coinhibitory roles in T-cells. CD161 has been extensively studied and characterized on subsets of T-cells that are MR1-restricted, IL-17 producing CD4^+^ (T_H_17 MAIT cells) and CD8^+^ T cells (Tc17 cells). Non-MAIT, MR1-independent CD161-expressing T-cells also exist and are characterized as generally effector memory cells with a stem cell like phenotype. Gene expression analysis of this enigmatic subset indicates a significant enhancement in the expression of cytotoxic granzyme molecules and innate like stress receptors in CD8^+^NK1.1^+^/CD8^+^CD161^+^ cells in comparison to CD8^+^ cells that do not express NK1.1 or CD161. First identified and studied in the context of viral infection, the role of CD8^+^CD161^+^ T-cells, especially in the context of tumor immunology, is still poorly understood. In this review, the functional characteristics of the CD161-expressing CD8^+^ T cell subset with respect to gene expression profile, cytotoxicity, and tissue homing properties are discussed, and application of this subset to immune responses against infectious disease and cancer is considered.

## Introduction

Establishment of long-lived memory T cell populations with enhanced cytotoxicity kinetics and that provide durable immunity against reinfection are critical to the cell mediated immune response (1). An intriguing subset of such memory T cells is identified by the expression of natural cytotoxicity receptor NK1.1 in mice and CD161 in humans and has been under investigation by several groups (2–9). In mice, the NK1.1 receptor is expressed on almost all NK cells and subsets of T cells including CD4^+^, CD8^+^, and NKT cells (5). While majority of the NK1.1^+^ T cells are CD4^+^, a significant proportion of viral antigen specific CD8^+^ T cells are reported to express NK cell markers in mice subjected to experimental viral infection (6, 10, 11). Unlike classical NKT cells, the CD8^+^NK1.1^+^ T cell subset is not only generated in NKT cell-deficient mice but also expresses a diverse polyclonal TCR repertoire suggesting these are not classical NKT cells (6). While some NK1.1^+^ CD8^+^ T cells are CD1d restricted, CD1d independent NK1.1^+^CD8^+^ cells with a memory phenotype have been found to be significantly increased in the liver and to persist following allo-HCT. Establishment of this TCRαβ^+^ T cell population derived from donor splenocytes and not bone marrow precursors could be abrogated by depletion of CD8^+^ cells but not NK1.1^+^ cells from the donor HCT inoculum, providing significant evidence that CD8^+^ T cells can acquire the expression of NK markers (12).

**Figure 1:**
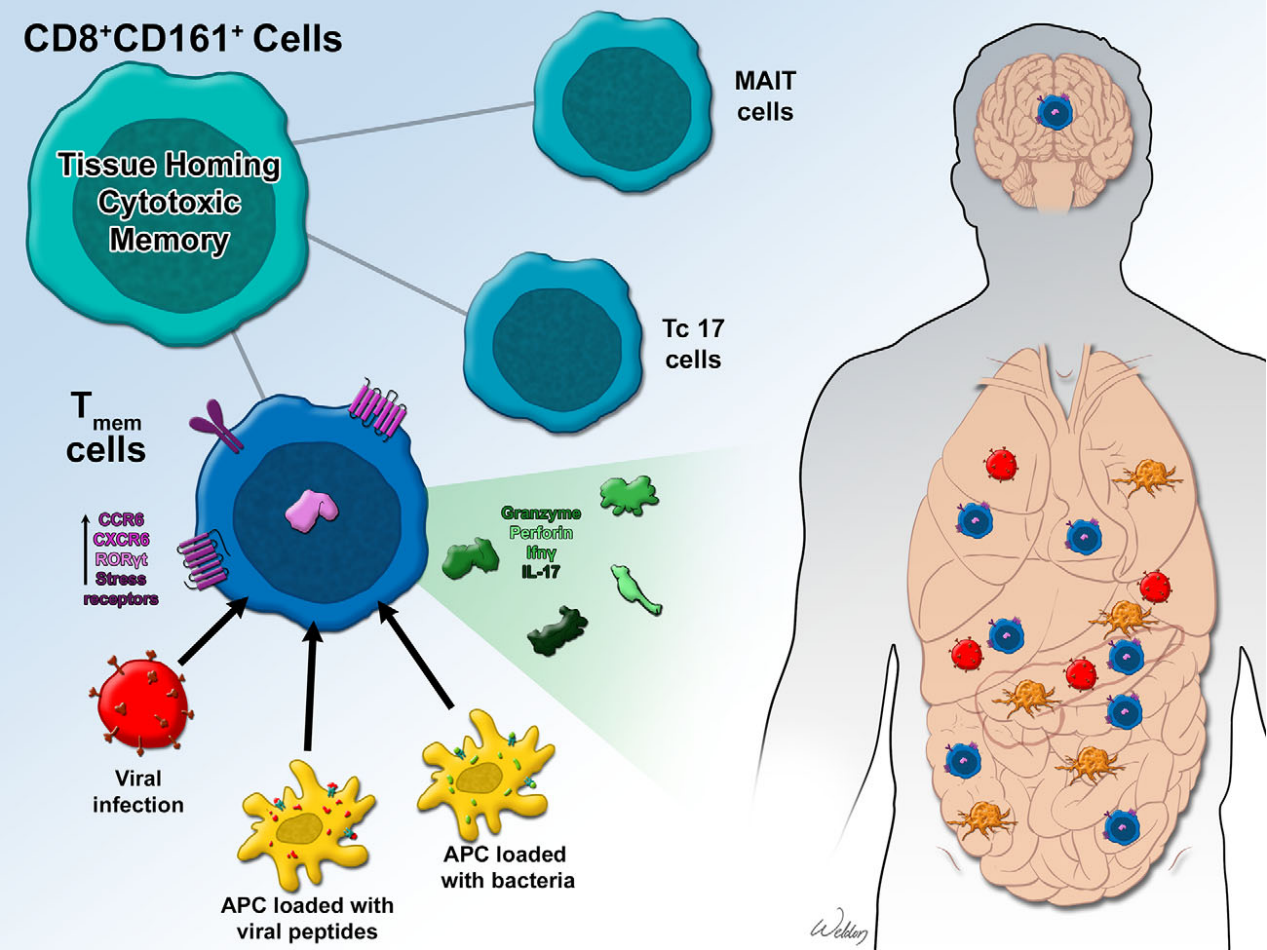
CD161-expressing CD8^+^ T cells are classified as tissue homing MAIT (Mucosal Associated Invariant T) cells, cytotoxic Tc17 cells, or T_mem_ (stem cell like memory) cells that display tissue homing and enhanced cytotoxic characteristics in addition to memory. T_mem_ cells respond to viral infections and can be activated by antigen presenting cells loaded with viral peptides or intracellular bacteria. In response to infection or antigenic stimulation, T_mem_ cells upregulate chemokine and stress receptors and secrete granzyme, INF-*γ*, and in some instances, IL-17. CD161^+^ T_mem_ cells home to vital organs like lungs, liver, gut, pancreas, and brain where they respond to viral infections and also display anti-tumor properties.

CD161, the human homolog of the mouse NK1.1 is expressed by NK cells and 24% of T cells including both γδ and αβ TCR-expressing subsets, NKT cells, monocytes, and dendritic cells (13, 14). CD161 expressing T cells exhibit varying levels of NK-cell like innate activity and are considered in some contexts as markers of “innateness” among T cells (15). Peripheral blood analysis of T cells from healthy donors indicates that CD161 is also preferentially expressed on memory T cell subsets (13). While CD4^+^ T cells express intermediate levels of CD161, CD8^+^ T cells may express CD161 at either intermediate or high levels (7, 9). CD161^high^ CD8^+^ T cells displaying a T_H_17 phenotype with upregulated expression of RORγt, CCR6, and IL18R are well characterized and described as Tc17 cells (**Figure 1**) (7). CD161^neg^ subsets exhibit naïve and central memory phenotypes while the CD161^high^ subset displays an effector memory phenotype (16). With no evidence of IL-17 secretion, CD161^int^ cells are reported to be a unique population of memory CD8^+^ cells with enhanced effector functions (7, 17, 18). Based on the expression of CCR7 and CD45RA, CD161^high^, CD161^int^, and CD161^neg^ subsets have been characterized as naïve, effector memory, and TEMRA (T effector memory with RA) phenotypes (18). While a majority of the CD161^high^ cells display a T_EM_ phenotype, CD161^neg^ cells display a T_CM_ phenotype, and CD161^int^ cells display both T_EM_ and T_EMRA_ phenotypes.

Previous studies have extensively characterized the shared pre-programmed phenotype of the MAIT and non-MAIT CD8^+^CD161^high^ T cells and their ability to respond to cytokine stimulation (9). MR1-restricted MAIT cells are the best characterized CD161^+^ CD8^+^ T cells and have been shown to detect a variety of microbes through the recognition of vitamin B metabolites presented by MR1 (19). Non-MAIT CD161^+^CD8^+^ T cells on the other hand exhibit specificity to viral antigens, are polyclonal in nature, and exhibit stem cell like memory phenotypes (**Figure 1**) (8). Apart from enhanced memory potential, low levels of PD-1 (exhaustion), CD161 expressing CD8^+^ T cells are also known to be tissue homing and demonstrate high levels of cytotoxicity against infectious agents, making them suitable therapeutic candidates against a wide range of infectious diseases and tumors (4, 7, 20). This review will focus on the expression of CD161 and the mouse homolog NK1.1 on polyclonal CD8^+^ T cells and discuss the biological significance of the expression of this receptor in disease.

## Cytotoxicity

### CD8^+^NK1.1^+^ T Cells in Mice Share Cytolytic Functions of Conventional CD8^+^ T Cells

Effective cellular immune responses rely substantially on the cytolytic ability of the responding immune cells and first line defenders. In this context, cytotoxic CD8^+^ T cells and NK cells play a critical role in providing durable and protective immune responses. Accordingly, research efforts are focused on the therapeutic potential of subsets that combine characteristics of both CD8^+^ T cells and NK cells (8). In mice, CD8^+^NK1.1^+^ T cells have been shown to share cytolytic functions of conventional CD8^+^ T cells with a T_H_1 skewed cytokine profile and lytic activity upon CD3 stimulation (21).

### Cytotoxicity of CD8^+^CD161^high^ Effector Memory T Cells Is Primarily Mediated Through Granzymes

In humans, the cytotoxic potential of different subsets of CD161^+^ expressing CD8^+^ T cells has not been clearly defined with reports of both high and low cytotoxic potential. CD8^+^CD161^high^ cells were once described as anergic subset of T cells with high levels of intracellular granzyme and perforin and no killing activity (5). In another report, CD161^high^ CD8^+^ T cells with a T_H_17 phenotype (Tc17 cells) displayed reduced cytotoxic potential compared to conventional effector CD8^+^ cells and were associated with low expression of granzyme B and perforin (8). Tc17 populations in mice also displayed limited cytolytic ability (22, 23). In contrast, another study reported that 29% of CD8^+^CD161^neg^, 56% of CD8^+^CD161^int^, and 88% of CD8^+^CD161^high^ cells stained positive for granzyme A suggesting high cytotoxic potential (5). CD161^high^ CD8^+^ T cells have also been reported to upregulate granzyme B and perforin and become highly cytotoxic upon activation (20) suggesting these are not similar to Tc17 cells but are more analogous to effector memory cells with stem cell like characteristics (3). We and others have also shown that, compared to the CD8^+^CD161^neg^ and CD8^+^CD161^int^ subsets, CD8^+^CD161^high^ cells produced significantly lower amounts of IFN-γ (5) suggesting that cytotoxicity may be mediated primarily through granzyme pathways.

## Tissue Homing

### Preprogrammed Expression of Tissue Resident Markers Enable Migration and Homing of CD8^+^CD161^high^ T Cells

Tissue resident innate and adaptive immune cells that do not recirculate in blood or lymphatics adopt a unique phenotype and contribute to barrier immunity, tissue homeostasis, and immune regulation (24). Studies on central and effector memory CD8^+^ T cell subsets distinguished by homing markers such as CCR7 and CD62L and detected in peripheral non-lymphoid organs have largely contributed to the concept of tissue-resident lymphocytes (16). Cytotoxic cells with tissue homing properties, especially to the mucosal tissues of the gastrointestinal tract, can act as a first line of defense at pathogen entry points and provide protective immune responses (18). Within the CD4^+^ and CD8^+^ T cell subsets, CD161^+^ cells make up more than half of T cells in the intestine (25). Among CD8^+^ T cells, tissue homing properties of subsets expressing CD161 have been well defined (7). Transendothelial migration of CD161^+^ CD8^+^ T cells without chemotactic stimuli suggests a tissue homing preference for these cells (14). CD8^+^ T cells expressing intermediate and high levels of CD161 are reported to also secrete high levels of IL-22, a cytokine involved in tissue repair and epithelial defense. Although MAIT cells were reported to be involved in tissue repair, recent studies on transcriptomic analysis of human and mouse MAIT cells identified a distinct tissue homing gene signature. Scratches on monolayers of colonic Caco2 cells were successfully closed by applying culture supernatants from MAIT cells activated by E. coli. MR1 blockade abrogated the effect confirming the TCR-dependent tissue repair potential of MAIT cells (26–30). CD161^high^ CD8^+^ MAIT cells are highly enriched in mucosal tissues and significantly upregulate chemokine receptors such as CXCR6 and CCR6 (31). In peripheral blood CD8^+^ cells, CD161 expression is associated with CXCR6, a chemokine receptor that binds CXCL16 constitutively expressed by the liver and respiratory tract (4, 32). CCR6^+^CD161^high^CD8^+^ cells have been detected in the naïve T cell population of cord blood suggesting pre-programmed expression of tissue resident markers enabling these cells to also home to non-lymphoid organs such as liver and gut (7). Previous studies have also shown that CD161 and CCR6 alone favor T cell migration and tissue homing (14, 33). Compared to the levels found in peripheral blood, CD161^high^CD8^+^ T cells are highly enriched in the gut and liver, while CD161^int^CD8^+^ cells are enriched in the colon. CD161^int^CD8^+^ cells also expressed CD103 and CD69, markers consistent with a tissue resident phenotype (18). CD161^high^ CD8^+^ MAIT cells with high expression of the multidrug resistance transporter ABCB1 are reported to preferentially home to intestine and liver and display an effector phenotype. Representing up to 45% of the liver lymphocytes, MAIT cells produce IFN-γ and granzyme B and upon stimulation with PMA/Ionomycin and secrete high levels of IL-17 (31). Thus, CD161 expressing CD8 T cells represent subsets of immune cells with therapeutic potential against tissue specific infections and diseases. Although there exists an overlap in the properties of CD161 expressing CD8^+^ T cell subsets, enhanced cytotoxicity, effector functions, tissue homing properties, survival and resistance to xenobiotics, make these cells key therapeutic effectors.

## CD8^+^NK1.1^+^/CD8^+^CD161^+^ T Cell Gene Expression Studies

### CD8^+^NK1.1^+^ Cells Express Innate Stress Receptors and Cytotoxic Granzymes

Gene expression profile analysis of CD1d independent, polyclonal CD8^+^NK1.1^+^ NKT-like cells, CD8^+^NK1.1^-^ cells, and conventional NK cells indicated that CD8^+^NK1.1^+^ cells showed a combination of T cell and NK cell markers, suggesting their potential to function as a kind of hybrid CTL/NK cell (34). Significantly elevated expression of pro-inflammatory cytokines, chemokines, chemokine receptors and other adhesion molecules indicate a distinct capacity to migrate, and elevated levels of cytotoxicity molecules involved in granule exocytosis suggest elevated killing potential (34). Gene expression analysis of antigen experienced CD8^+^NK1.1^+^ cells revealed a significant upregulation of cytotoxic granzymes F, D, G, C, B, A, and N and innate like stress receptors in comparison to the CD8^+^NK1.1^-^ subset.

### CD8^+^CD161^+^ Cells Express Chemokine, Tissue Homing, Cytotoxic, and Innate Stress Receptors

Previous gene expression studies of CD8^+^CD161^+^ Tc17 cells revealed upregulation of several T_H_17 related genes including RORγt, CYP1B1, and the chemokine receptors IL23R, CXCR6, and IL8R (7, 35). CCR7 was downregulated among the CD161^+^ cells (7). Gene expression analysis of CD161^int^ cells from adult circulating peripheral blood and naïve umbilical cord blood revealed significant upregulation of IL18R, CXCR6, MDR1, and PLZF indicating a preprogrammed phenotype with tissue homing properties (18). Expression of MDR1 on the CD161^high^ and CD161^int^ subsets enables survival of these subsets in gastrointestinal tissues exposed to xenobiotics (18). CD161^int^ subsets also showed significantly elevated levels of granzyme B and perforin compared to the CD161^neg^ subsets. CD161^high^ MAIT cells on the other hand are potent producers of IFN-γ (9, 18). Different subsets of CD8^+^CD161^+^ T cells including MAIT, Tc17, and non-MAIT effectors show overlap in gene expression profiles suggesting enhanced tissue homing and migratory potential of these cells is facilitated by the combined action of different subsets.

## Molecular Mechanisms of NK1.1/CD161 Ligand Interactions

In mice, NK1.1 belongs to the family of NKRP1 receptors, a family of disulfide linked homodimers that interact with C-type lectin related (Clr) molecules (13). NKRP1B and NKRP1D are inhibitory receptors and recognize Clr-b (encoded by Clec2d) while the activating receptor NKRP1F binds to Clr-g (36, 37).

### CD161 Engagement With Its Ligand LLT1 Can Be Both Costimulatory and Inhibitory

In humans, the CD161 receptor KLRB1 is expressed on NK and T cells and interacts with the ligands Clec2/Lectin like transcript-1 (LLT1) and the lesser characterized PILAR (proliferation induced lymphocyte receptor) (38–40). PILAR is known to modulate T cell expansion and acts as a survival signal for CD161-expressing naïve and activated T cells (41). Restricted to hematopoietic cells, LLT1 is not expressed on the surface of resting PBMCs but can be transiently expressed on activated B cells, dendritic cells, T cells and NK cells (42). Elevated expression of LLT1 has been reported on germinal center B cells in tonsils and lymph nodes and B cell derived lymphomas (43). In NK cells, the interaction of LLT1 with the CD161 receptor is described as inhibitory (13, 39, 44) while in T cells, CD161 engagement with LLT1 is considered both costimulatory (39, 45) and inhibitory (38, 46). CD161 receptor engagement with the ligand LLT1 was not sufficient to trigger IFN-γ production among T cells unless simultaneously engaged with CD3 (39). LLT1 interaction with CD161 did not modulate degranulation in CD8 T cells but partially inhibited TNF-α production (38). Since CD161 is not known to possess any characterized signaling motifs, additional studies will be required to better understand the true consequences of this ligand/receptor interaction (39).

### CD161 Intracellular Signaling in CD8^+^ T Cells Is Not Well Defined

In NK cells, CD161 has been shown to directly interact with acid sphingomyelinase (ASM) resulting in intracellular AKT signaling and regulation of NK cell function (47). In T cells however, especially CD8^+^ T cells, CD161 signaling is not very well defined. In CD4^+^ CD161^+^ Th17 cells, CD161 is shown to interact with the surface receptor CD39 to further amplify ASM-mediated mTOR and STAT3 signals driving Th17 expansion (48, 49). Further studies are warranted in understanding the signaling events among non-Th17 CD8^+^ CD161^+^ T cells.

## Role of CD8^+^NK1.1^+^/CD8^+^CD161^+^ in Infection and Immunity

In the infectious disease literature, CD8^+^NK1.1^+^ cells and the analogous human CD8^+^CD161^+^ cells are described as highly cytotoxic memory T cells with antiviral specificity (6–8, 11, 18).

### CD8^+^NK1.1^+^ T Cells Are Protective Against Viral Infections and Intracellular Pathogens

In previous studies, we observed a significant upregulation in the number of peripheral CD8^+^NK1.1^+^ cells following administration of a T_H_1 polarized dendritic cell vaccine (50–53). In mice, TGF-β is reported to repress the differentiation of NK1.1^+^ T cells from CD8^+^ T cells. However, upon bacterial or viral infection a fraction of CD8^+^ T cells have been shown to escape TGF-β control during priming and acquire NK1.1 expression. In an LCMV infection model, unlike the CD8^+^NK1.1^neg^ subset, the CD8^+^NK1.1^+^ cells underwent delayed contraction and apoptosis and provided prolonged pathogen-specific reactivity by producing IFN-γ and granzyme B (54). Endowed with innate immunity features and contributing to the adaptive immune responses, these CD8^+^NK1.1^+^ cells not only cleared the initial microbial and viral infections but also offered protection against reinfection. In a study involving lethal *Listeria* infection, CD8^+^NK1.1^+^ cells provided rapid innate immune responses characterized by early, antigen-independent IFN-γ production, granzyme B expression, degranulation, and protection against re-exposure (55). In a separate study, CD8^+^NK1.1^+^ T cells were shown to comprise 10% of total CD8^+^ T cells in the lungs and offer durable protection at ten days after primary influenza infection (11). These cells were elevated in number in CD1d^-/-^ mice suggesting they are not NKT cells but a distinct population in which NK1.1 may modulate effector functions of activated antigen experienced CD8^+^ T cells. CD8^+^NK1.1^+^ cells described as Tc17 cells were also highly protective against lethal influenza infection (23). In intracellular parasite infection models, CD1d-independent CD8^+^NK1.1^+^ T cells have been shown to play a protective role against the liver stage of *Plasmodium yoelii* infection (56). A significant increase in the number of splenic antigen experienced, activated CD8^+^NK1.1^+^ T cells was also seen during the acute stage of *Plasmodium chabaudi* infection (57). These studies suggest that in murine models, CD8^+^NK1.1^+^ T cells are protective against viral infections and intracellular pathogens. Antigen dependent activation leads to an enhanced proliferation of these cells and upregulation of innate stress receptors, cytotoxic molecules resulting in durable protective responses against reinfection and improved disease-free survival.

### CD8^+^CD161^+^ Cells Offer Pathogen Immunity, Specifically to Viral Infection

In humans, CD161 has been reported as a marker for long lived memory CD4^+^ T cells. It was reported that the proportion of influenza specific CD4^+^CD161^+^ T cells was more highly elevated at two years post immunization than four weeks post immunization, suggesting that CD161 is a marker of long-term memory among T cells (58). Several groups have reported the role of CD8^+^CD161^+^ cells in pathogen immunity, specifically immunity to viral infection. Enrichment of CD161^+^ cells was seen in the liver in response to infection and non-alcoholic steatohepatitis (7). CD8^+^CD161^+^ cells specific for hepatitis C virus (HCV) and hepatitis B virus (HBV) were reported earlier (4). T_H_17 cells responding to HCV specific peptides have been reported (7, 59). CD161^+^ MAIT cells on the other hand are reported to be responsive to bacterial infections (19). CD161^high^CD8^+^ T cells specific for EBV, CMV, or influenza encompassed IL18Ra^high^, IL7, and IL15 responding memory cells expressing higher levels of anti-apoptotic molecules and high drug efflux capacity (3) suggesting these cells can survive hostile inflammatory conditions leading to pathogenesis of tissues such as in inflamed CNS.

## IL-17 Producing CD161^+^ T Cells Implicated in Auto-Immune Diseases

CD161 expressing T cells, specifically the IL-17 producing subset, have been implicated in auto-immune diseases like psoriasis, Crohn’s disease, rheumatoid arthritis, and multiple sclerosis (7, 60–64). A subset of CD8^+^CD161^int^ cells with elevated expression of granzyme B and perforin have been shown to cross the blood brain barrier and are enriched in MS lesions (17). While enriched in the CNS, CD8^+^CD161^+^ cells were reduced in number in the peripheral blood in MS patients in comparison to healthy adults. In MS brain infiltrates, 10% of all CD8^+^ T cells were IFN-γ producing CD161^+^ cells that also secreted IL-17 and IL-22 and contributed to the pathogenesis of the disease (64). Activation induced expansion of CD161 cells and the implication of CD161 polymorphism in MS suggests potential therapeutic modulation of these cells in disease conditions mediated or ameliorated by CD8^+^ T-cells (40, 65, 66). In SLE, a disease in which CD8^+^ cells play a relatively minor role in pathogenesis, reduced CD161 expression on CD8^+^ T cells and NKT cells was noted in patients with advanced disease (67).

## Roles of CD8^+^NK1.1^+^/CD8^+^CD161^+^ in Tumor Immunology

### CD8^+^NK1.1^+^ T Cells Offer Protection and Improve Survival Against Multiple Murine Tumor Malignancies

Although the role of NK1.1^+^ T cells is defined in the context of viral infection, little is known about any potential role for this receptor on CD8^+^ T cells in the context of cancer. In mice, *in vitro* expansion of highly cytotoxic CD8^+^NK1.1^+^ T cells derived from bone marrow, spleen, and thymus have been shown to mediate strong anti-leukemia effects without GVHD after allogeneic transplantation (68, 69). In a model of murine pancreatic cancer, NKT cells have been shown to offer protection by modulating tumor associated macrophages to drive a T_H_1 adaptive immune response (70). In another study, the tumoricidal effects of the CD8^+^NK1.1^+^ cells derived from OT-1 mice were significantly higher than that of the CD8^+^NK1.1^neg^ equivalent population (71). In a B16 lung metastatic *in vivo* model, adoptive transfer of CD8^+^NK1.1^+^ NKT-like cells significantly inhibited metastasis and improved survival in comparison to adoptive transfer of CD8^+^NK1.1^neg^ cells or conventional NK cells (34). In *in vitro* killing assays, CD8^+^NK1.1^+^ NKT-like cells exerted cytotoxicity against tumor cells and MDSCs (myeloid derived suppressor cells) through a granzyme B-mediated granule exocytosis pathway. Granzyme B inhibitors suppressed the cytotoxic effects while treatment of the cells with anti-FasL, anti-TRAIL, or anti-IFN-*γ* antibodies did not (34). In a previous study, we demonstrated that adoptively transferred CD8^+^NK1.1^+^ cells offered durable protection against murine PDAC (pancreatic ductal adenocarcinoma) and improved survival (53). These cells were present nine months after initial antigen exposure and were potent in clearing tumors. Gene expression analysis revealed that these CD8^+^NK1.1^+^ cells exhibited significantly elevated levels of cytotoxic molecules and stress receptors. These studies highlight the role of CD8^+^NK1.1^+^ cells in generating durable anti-tumor responses against murine tumors suggesting their potential in cell based therapeutics. Enhanced cytotoxicity and memory characteristics of these cells make them ideal candidates for cell based therapies against aggressive malignancies.

### CD161 Expression on T Cells Results in Both Favorable and Unfavorable Outcomes Against Tumors

In humans, there are limited studies defining the role of CD161 expression on CD8^+^ T cells in tumor immunology with reports suggesting both favorable and unfavorable outcomes. In a cancer- wide genome analysis of prognostic gene signatures, KLRB1, the gene encoding CD161, was identified as most frequently associated with favorable outcomes against several indications including bladder, breast, colon, prostate cancers, melanoma, lung adenocarcinoma, multiple myeloma, glioma, and neuroblastoma to name a few (72, 73). Transcription of KLRB1 was suppressed in 68% of NSCLC (non-small cell lung cancer) and 57% of esophageal squamous-cell carcinoma patients indicating that CD161 can be a predictive marker in these indications (74). In NSCLC, interaction of CD161 expressing tumor infiltrating CD4^+^ and CD8^+^ T cells with LLT1-expressing germinal center B cells within tumor microenvironment tertiary lymphoid structures resulted in improved survival (73). In the CD4^+^ T cells, genes associated with KLRB1 expression were CCR2, CCL4, and GZMA, markers of a T_H_1 polarized effector phenotype (73). Similarly, in oropharyngeal cancer, IFN-γ and IL-17 producing CD161^+^ tumor infiltrating T cells were associated with better tumor control (75). In head and neck cancer, CD161 expression was significantly down modulated in the peripheral blood compared to peripheral blood from healthy controls and in tumor infiltrating T_H_17 cells as a postulated immune escape mechanism induced by the tumor milieu (41). In a recent study of hepatocellular carcinoma (HCC), infiltration of CD8^+^ PD-1^+^ CD161^+^T cells into the tumor microenvironment represented reactivated cytotoxic cells with proliferative and not exhaustive characteristics and correlated with better prognosis. Co-expression of IL-7R and enhanced expressions of IL-2, TNF- α and perforin mediated the maintenance of proliferative phenotype (76). These studies highlight the therapeutic potential of CD8^+^CD161^+^ T cells as prognostic biomarkers and/or candidates for cell based therapies. Enhanced cytotoxicity, tissue homing and memory characteristics make them suitable candidates against aggressive malignancies (**Table 1**).

**Table 1:** Roles of CD8+NK1.1+/CD8+CD161+ Cells in Infection and Immunity.

Species	T cell subset	Disease/Model	Mechanism of action	Reference
		**Viral/parasitic infections**		
Murine	CD8+NK1.1+	Influenza infection	10% of the lung CD8+T cells upregulate NK cell receptor NK1.1+ upon infection and offer protection	Kambayashi et al. (11)
		Influenza infection	Protection by Tc17 effector cells against influenza infection is IFN-γ dependent and accompanied by greater neutrophil influx into the lung	Hamada et al. (23)
		LCMV infection	CD8+NK1.1+ T cells escape TGF-beta control, resulting in delayed contraction and apoptosis and early pathogen response	Ruiz et al. (54)
		Listeria infection	CD8+NK1.1+ T cells offer protection by early antigen independent innate immune responses mediated by IFN-γ production and GzmB expression	Seregin et al. (55)
		Malaria	Increase in the liver cell numbers of CD8+NK1.1+ cells offer protection against liver stage *Plasmodium yoelii* infection	Pied et al. (56)
		Malaria	Significant increase in splenic antigen experienced, activated CD8+NK1.1+ T cells offer protection against acute *Plasmodium chabaudi* infection	Muxel et al. (57)
Human	CD8+CD161+	Bacterial infections	Enhanced migration and enrichment of IFN-γ secreting MAIT cells at the sites of infection helps clear the pathogen	Le Bourhis et al. (19)
		EBV/CMV/influenza	Self renewing, antigen specific CD8+CD161high memory cells express anti-apoptotic molecules and survive cytotoxic chemotherapy	Turtle et al. (3)
		HCV/HBV	Antigen specific CD8+CD161+ T cells secrete pro-inflammatory cytokines IFN-γ and TNF-α and offer protection	Northfield et al. (4)
		Viral infection	CD8+CD161+ T cells markedly enriched in the liver coexpressed IL-17 with high levels of IFN-γ and/or IL-22 and offer protection against viral infections.	Billerbeck et al. (7)
		**Tumor biology**		
Murine	CD8+NK1.1+	Leukemia/lymphoma	Expansion of cytolytic NKT cells producing IFN-γ limits GVHD in leukemia	Baker et al., Verneris et al. (68, 69)
		Melanoma	NKT like CD8+NK1.1+ T cells exert cytotoxicity against tumor cells and MDSCs, inhibit metastasis and improve survival	Li et al. (34)
		Murine Pancreatic ductal Adenocarcinoma	Adoptive transfer of antigen experienced CD8+NK1.1 cells offer anti-tumor protection	Konduri et al. (53)
		Pancreatic Cancer	Anti-tumor effects of NKT cells is dependent upon tumor associated macrophage mediated TH1 adaptive immune response	Janakiram et al. (70)
Human	CD8+CD161+	Head and Neck cancer	In HNSCC, immune evasion is mediated by down regulation of CD161 on Th17 cells in peripheral blood, primary tumor tissue, and lymph nodes	Kesselring et al. (41)
		HPV-Oropharyngeal squamous cell cancer	Antigen specific CD161+ T cells produce IL-17 and IFN-γ in the type-1 oriented tumor microenvironment resulting in reduced tumor burden and improved overall survival	Welters et al. (75)
		Hepatocellular Carcinoma	Co-expression of CD161 and IL-7R helps maintain proliferation of CD8+PD-1+ T cells partly through enhanced expression of IL-2, TNF-α, and perforin resulting in better prognosis	Li et al. (76)
		Lymphoma	In transplant setting, host NKT cells prevented lethal GVHD while perforin producing CD8+ T cells offered graft antitumor activity	Pillai et al. (77)
		Lymphoma	IL21 promoted expansion of Th1 skewed cytotoxic CAR NKT cells offering protection	Ngai et al. (78)
		Multiple tumors	KLRB1, the gene encodign CD161 is associated with favorable outcomes against multiple tumor models	Gentles et al., Braud et al. (72, 73)
		Multiple tumors	The potential immunoregulatory role of CD4+ CD161+ cells is mediated through soluble factors, high IL-10, IL-4, and TGF-β resulting in disease progression	Iliopoulou et al. (79)
		NSCLC	Tumor infiltrating CD8+CD161+ interact with LLT1 expressing germinal center B cells within TME resulting in improved survival	Braud et al. (73)
		Pediatric Leukemia	iNKT cell subsets in hHSCT recipients contribute to the maintenance of the remission state, possibly through the provision of antitumor cytokine IFN-γ.	de Lalla et al. (80)
		**Autoimmunity**		
Human	CD8+CD161+	Autoimmune diseases	IL-17 producing Th17 cells mediate tissue inflammation and autoimmune progression in gut, joints, and brain by secreting inflammatory cytokines like IL-23	Cosmi et al., Kleinschek et al., Lock et al., Tzartos et al., Annibali et al. (60–64)
		Systemic Lupus Erythematosus	Abnormalities in the frequencies and levels of CD161 expression on CD8+ T cells and NKT cells correlate to the pathogenesis of SLE.	Park et al. (67)

### CD161 Expression Correlates With Immune Regulatory Functions

In other studies, CD161 expression has been described as a negative prognostic marker. In a previous study, analysis of several different malignancies showed a significant increase in the number of CD161 expressing CD4^+^ T cells in the peripheral blood of cancer patients compared to healthy individuals, and this increase was positively correlated with disease stage. The potential immunoregulatory role of CD4^+^ CD161^+^ cells was mediated through soluble factors, mainly through high IL-10, IL-4, and TGF- β (79). In T cell lymphoblastic leukemia, aberrant expression of CD161 and other NK cell markers like CD56 and CD16 on T cells was correlated with disease progression (81). In T cell pro-lymphocytic leukemia (T-PLL), aberrant CD161 expression on T cells was not associated with other NK cell markers suggesting the usefulness of this unique phenotype as a diagnostic marker (82). These studies suggest that the expression of CD161 on T cells can serve both activating and inhibitory roles in tumor progression.

The natural anti-tumor effector properties of CD1d restricted NKT cells have been effectively used to treat lymphomas without associated GVHD. Peripheral blood NKTs also offered long term remission against pediatric leukemia (77, 80). Ex vivo expanded CAR NKTs in the presence of IL-21 promoted expansion of highly cytotoxic T_H_1 polarized cells that enabled long term survival of lymphoma bearing mice (78). However, the potential applicability of CD161 expressing CD8^+^ CAR T cells in the solid tumor setting has not yet been evaluated.

## Conclusions and Future Work

CD8^+^CD161^+^ T cells and their murine counterparts CD8^+^NK1.1^+^ T cells exhibit elevated cytotoxic potential, characteristics of long-term memory, drug–effluxing capacity, and extended survival and, as such, are potential candidates for adoptive cell therapy to treat pathologic indications (3, 58). Tissue homing properties of these cells to mucosal surfaces may mediate protection at pathogen sites of entry (18). Gene expression analysis indicates enhanced expression levels of granzyme, perforin, and innate-like receptors among these cells when activated in comparison to NK1.1^neg^/CD161^neg^ counterparts. Although studied extensively in the context of viral infection, there is limited information on the homeostatic function of this cell subset in cancer progression. With confounding reports of protective and inhibitory potential, it is imperative that they are tested for their efficacy under specific conditions. The effector functions of these cells operate by both cytolytic and noncytolytic mechanisms. In the case of LCMV infection, viral load is controlled by cytotoxic granzymes and perforin secreted by these cells, while antiviral activity against HBV and HCV is regulated principally by IFN-γ secretion. Protective effects of these cells against murine influenza is also driven by IFN-γ secretion. Thus, cytolytic and non-cytolytic protective effects of these cells depend upon the pathogenicity of infection and the tissue environment (**Table 1**). Little is known about the cascade of signaling events that lead to the effector functions of CD161 activation. Further work is needed to delineate the role of costimulatory and signaling molecules during CD161 engagement. When simultaneously stimulated with CD3, ligation of CD161 on CD8^+^ T cells serves as a co-stimulatory molecule for IFN-γ production as seen during pathogenesis of multiple sclerosis. Ligand interactions play an important role in the effector functions of this subset, and it will be interesting to test the cytotoxic efficacy of these cells against tumors expressing LLT1. Nonetheless, the sum total of the data indicated that CD8^+^CD161^+^ T cells are critical effector memory cytolytic effectors that are T_H_1 polarized, tissue homing, and primarily of anti-viral specificity. Future investigations into the biology of these cells will focus on signaling events downstream of CD161 ligation but will also necessarily look upstream at the signals, environmental conditions, and APC subsets present at the time of priming.

## Author Contributions

VK, DO-S, JV-P, MH, JL, and WD all collaborated in the writing and editing of this manuscript. SW provided the original artwork. All authors contributed to the article and approved the submitted version.

## Funding

This work was funded in part by NIH R01 AI127387 (to WD).

## Conflict of Interest

Institutional policy requires VK, MH, and WD to declare their ownership stakes in Diakonos Research, Ltd.

The remaining authors declare that the research was conducted in the absence of any commercial or financial relationships that could be construed as a potential conflict of interest.
